# Communication, Cooperation, and Social Interactions: a Report from the Third Young Microbiologists Symposium on Microbe Signalling, Organisation, and Pathogenesis

**DOI:** 10.1128/JB.02029-14

**Published:** 2014-10

**Authors:** Delphine L. Caly, Sarah J. Coulthurst, Shi-qi An, Sophie Helaine, Jacob G. Malone, Robert P. Ryan

**Affiliations:** a ProBioGEM-Polytech-lille, Université Lille 1, Villeneuve d'Ascq, Lille, France; b Division of Molecular Microbiology, Colleges of Life Sciences, University of Dundee, Dundee, United Kingdom; c Section of Microbiology, Medical Research Council Centre for Molecular Bacteriology and Infection, Imperial College London, London, United Kingdom; d University of East Anglia/John Innes Centre, Norwich, United Kingdom

## Abstract

The third Young Microbiologists Symposium took place on the vibrant campus of the University of Dundee, Scotland, from the 2nd to 3rd of June 2014. The symposium attracted over 150 microbiologists from 17 different countries. The significant characteristic of this meeting was that it was specifically aimed at providing a forum for junior scientists to present their work. The meeting was supported by the Society for General Microbiology and the American Society for Microbiology, with further sponsorship from the European Molecular Biology Organization, the Federation of European Microbiological Societies, and The Royal Society of Edinburgh. In this report, we highlight some themes that emerged from the many exciting talks and poster presentations given by the young and talented microbiologists in the area of microbial gene expression, regulation, biogenesis, pathogenicity, and host interaction.

## INTRODUCTION

Microbiology has already had a long and rich history, initially centered on the causes of infectious diseases but now implicated in almost every facet of life. With the genomics revolution and the introduction of molecular biology, our understanding of microbial life has changed rapidly in a very short period of time. While much has been learned, many questions regarding the mechanistic basis of regulation, physiology, and, importantly, pathogenicity and host interaction are still to be answered.

On the 2nd to 3rd of June 2014, over 150 scientists dedicated to this pursuit descended on the University of Dundee for the third Young Microbiologists Symposium (YMS), consisting of 34 talks and more than 80 posters. Like the previous two meetings, this was held under the inclusive title “Microbe Signalling, Organisation and Pathogenesis” (1, 2). The meeting was cochaired by Sarah Coulthurst (University of Dundee) and Robert Ryan (University of Dundee). The key aspect of this meeting was that it was specifically designed to provide a forum for junior scientists to present their research and network with their peers. For each of the broad theme-based sessions, chaired by postdocs or new principal investigators (PIs), the first lecture was given by an eminent academic who has made a substantial contribution to the area, while the majority of the talks were given by young researchers from leading laboratories around the world.

The meeting highlighted the extraordinary range of functions associated with bacterial life, emphasized recent discoveries regarding the diverse array of signaling and regulatory processes in bacterial development and virulence, described several novel technologies for studying bacterial behavior, and underlined our growing understanding of bacterial interspecies and even interkingdom communication. In this Meeting Review, we give a brief summation of the work presented during the meeting, as described in both oral and poster presentations. Due to space limitations, we can summarize only some of the highlights of the meeting, and we apologize to participants whose excellent work could not be mentioned here.

## SMALL REGULATORY RNAs, RNA-BINDING PROTEINS, AND ENERGY-DEPENDENT PROTEOLYSIS

Susan Gottesman (National Cancer Institute, USA) delivered the American Society for Microbiology-sponsored lecture, entitled “On and Off Switches for Bacterial Small RNAs.” Here she focused on discussing how the synthesis of many of Escherichia coli small RNAs (sRNAs) are regulated (the “on” switch) and how Hfq, an RNA chaperone protein, acts to regulate the stability of many of these sRNAs (the “off” switch). Susan started by highlighting some of the landmark studies describing bacterial sRNA discovery, to which she has made several major contributions. She explained that although regulatory RNAs have been a major topic of interest in eukaryotic cells, studies of bacterial sRNAs have been equally exciting, with a wide range of bacteria now having been shown to utilize these regulatory molecules to control many critical cellular processes (3).

**FIG 1:**
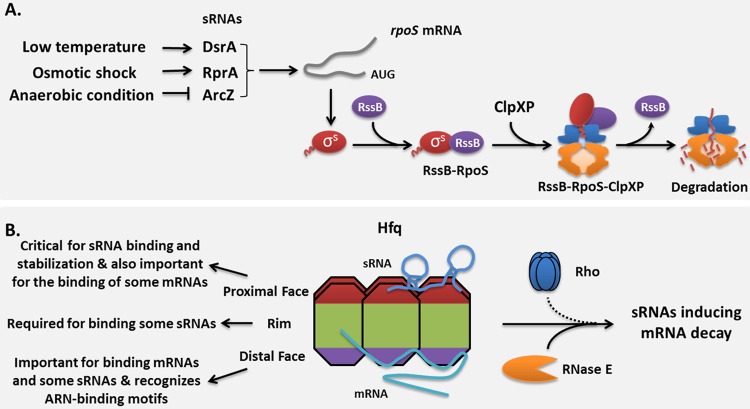
Small RNA regulatory circuits. (A) Small RNAs are synthesized in response to different stresses, allowing the bacterium to integrate and respond to numerous stress signals for control of *rpoS* translation. (B) Bacterial sRNAs use the Hfq chaperone to mediate pairing with and regulation of mRNA stability and translation. For further details, see references 8 and 10.

Susan's laboratory first encountered small RNAs when studying the synthesis and regulation of substrates for energy-dependent proteolysis in E. coli. The work on sRNAs in her group was stimulated by the fact that RpoS, the stress sigma factor of E. coli, is regulated by sRNAs at the posttranscriptional level. She described how RpoS is rapidly degraded only during exponential growth by the ClpXP protease (4). This process required the response regulator RssB as an adaptor protein for RpoS degradation; RssB appeared to affect the degradation of only RpoS and not of other ClpXP substrates both *in vivo* and *in vitro* (5). In the same study, Susan and her colleagues identified several small anti-adaptor proteins that were synthesized in response to specific environmental signals and affected the ability of RssB to deliver RpoS to the ClpXP protease (5). Interestingly, the group demonstrated that, in addition to being regulated by this mechanism, RpoS was positively regulated by multiple sRNAs at the transcriptional level (4). DsrA, one such sRNA, is produced during low-temperature growth, consistent with one of the many conditions required for RpoS expression (6). DsrA acts to positively regulate the synthesis of RpoS through base pairing with a sequence in the 5′ untranslated region of the *rpoS* mRNA (6). Another sRNA, RprA, also stimulates the synthesis of RpoS through a pairing mechanism similar to that identified for DsrA (7). However, RprA is regulated by a two-component regulatory system that is responsive to cell surface conditions instead of low temperature (7). In addition, a third sRNA identified, called ArcZ, also acts to positively modulate RpoS and is controlled by regulators involved in sensing aerobic/anaerobic growth (Fig. 1) (8). Intriguingly, all three sRNAs also negatively regulate other mRNAs, providing an even more complex regulatory network. These findings helped to illuminate how these regulators interact with and affect each other and assemble the global control circuits.

In the second part of the lecture, Susan discussed her group's focus on a large group of bacterial sRNAs, which includes RprA, ArcZ, and DsrA, that use the Hfq chaperone to mediate pairing with and regulation of mRNA stability and translation. Susan explained how this work was ignited via a genome-wide collaborative study examining the large number of sRNAs that bind the RNA chaperone Hfq. This led to identification of yet more sRNAs, although less conserved, in E. coli that were not envisaged. Combining this observation with those of other studies reveals and confirms more than 80 sRNAs in E. coli, demonstrating the complex sRNA regulatory circuits that are in play in many bacteria (9). In addition to defining targets, they extended their study to examine the variety of pathways that lead to sRNA-mediated degradation of specific mRNA targets and how Hfq engages the various proteins associated with these pathways. Their recent *in vivo* analysis of multiple *hfq* mutants revealed differential functions of specific amino acid substitutions, and phenotypic assays further confirmed the essential role for the proximal face of Hfq, which is involved in sRNA binding (10). They also demonstrated that the “rim” of the Hfq hexamer is important for regulation and that individual sRNA-mRNA pairs have different sensitivities to *hfq* mutants (Fig. 1) (10). Taken together, this work revealed an unexpected complexity in how Hfq modulates sRNA-based regulation. Building on this point, Susan further explained how these sRNAs induced mRNA decay and the decoupling of transcription and translation involving RNase E and Rho and suggested that prokaryotes and eukaryotes have evolved different machineries to process and present regulatory RNAs, but these sRNAs facilitate similar outcomes to help them to adjust to specific physiological requirements (11).

To finish, Susan concluded that although sRNAs have been identified in a wide range of bacteria to play critical regulatory roles in many processes and the methods for discovery are advancing rapidly, many questions are still left unanswered.

## REGULATORY NETWORKS, SIGNAL TRANSDUCTION, AND INTRACELLULAR SIGNALING

**FIG 2:**
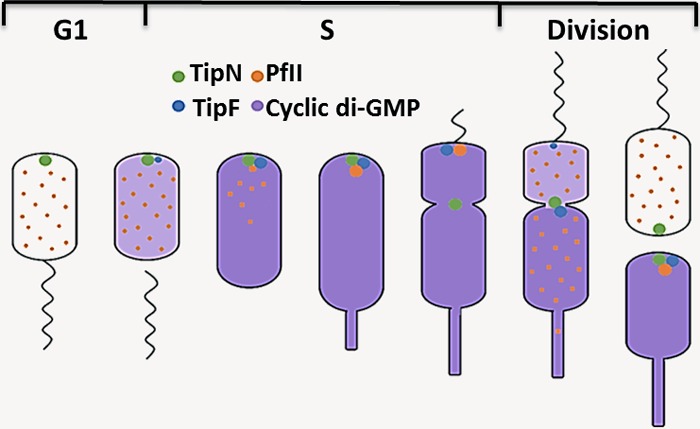
Model for TipN/TipF flagellum localization. TipN localizes to the site of cytokinesis and recruits newly synthesized TipF–c-di-GMP when the cellular c-di-GMP level rises at the G_1_- to S-phase transition. TipF-c-di-GMP then recruits the flagellar localization factor PflI, which in turn recruits the initial flagellar components and initiates flagellum formation. TipN subsequently relocates to the site of cell division, and once flagellum formation is completed, PflI delocalizes. Cell division leads to asymmetrical concentrations of c-di-GMP in the stalked and swarmer cells, leading to TipF degradation in the swarmer cell (c-di-GMP-low) and TipN-TipF localization in the stalked cell (c-di-GMP-high). (Adapted from reference 12 with the permission of the publisher.)

The Gene Regulation and Intracellular Signaling session showcased a diverse mix of research, addressing both the specifics of second-messenger signaling pathways and fundamental aspects of transcriptional regulation. A fascinating example of the former was presented by Urs Jenal (University of Basel), who gave the opening EMBO Lecture on the regulation of Caulobacter crescentus development and cell cycle control by cyclic di-GMP (c-di-GMP). Urs explained his recent collaborative work with Patrick Viollier to establish a mechanism for c-di-GMP control of flagellated, swarmer pole biogenesis (12) (Fig. 2). The flagellar assembly factor TipF is a degenerate phosphodiesterase that binds c-di-GMP when levels rise during the G_1_- to S-phase transition and localizes to the new cell pole, marked by TipN deposition at the site of cytokinesis. c-di-GMP-bound TipF in turn recruits FliG (via PflI) to the cell pole, initiating flagellum formation. The stability of TipF depends on c-di-GMP binding, so as the c-di-GMP level drops upon cell division, TipF is degraded and flagellum formation is suspended until the next cell cycle (12). Urs also presented some intriguing new research from his group on the discovery and characterization of several novel c-di-GMP binding proteins in C. crescentus.

Once again, c-di-GMP signaling was well represented at the YMS. Liz Sockett (University of Nottingham) presented her work on the control of Bdellovibrio prey invasion (described in more detail below), Natalia Tschowri (HU, University of Berlin) discussed her exciting findings on the role of c-di-GMP signaling in Streptomyces venezuelae development, and Jake Malone (UEA/John Innes Centre) presented his group's research on c-di-GMP signaling during Pseudomonas fluorescens plant colonization. Jake's lab has identified a composite GGDEF/EAL protein (RccA) that plays an important role in wheat rhizosphere colonization. RccA works in tandem with the transcriptional regulator RccR to sense and respond to different environmental carbon sources. The coordinated activities of RccA and RccR subsequently control cell motility and growth rate, with corresponding effects on rhizosphere colonization efficiency. Continuing the theme of second-messenger signaling, Rebecca Corrigan (Imperial College London) discussed her exhaustive analysis of the Staphylococcus aureus c-di-AMP network. Following her earlier work in the Gründling lab that identified c-di-AMP signaling in S. aureus (13), Rebecca screened the entire S. aureus proteome for c-di-AMP binding proteins. Affinity pulldown experiments and subsequent bioinformatic analysis identified the potassium channel regulator KtrA and a second protein involved in ion uptake, CpaA. In parallel, a further two proteins were identified by DRaCALA screening (14). The proteins PstA, a nitrogen regulator, and KdpD, a histidine kinase, control the expression of potassium transporter genes, further establishing c-di-AMP as a master regulator of potassium homeostasis in S. aureus (14).

Finally, David Grainger (University of Birmingham) presented a very different perspective with his examination of transcriptional regulation in Escherichia coli, a well-established model organism for bacterial genetics. David's lab has established that correct transcription of the AT-rich *ehxCABD* operon relies on the extensive masking of spurious promoters throughout the operon by the histone-like nucleoid structuring (H-NS) protein (15). Extending their analysis to the whole E. coli genome, David and coworkers showed that this H-NS-mediated transcriptional suppression is widespread, with 46% of suppressed transcripts being intragenic in origin and many more H-NS-repressed promoters being located upstream of noncoding RNAs (16). This work both refines the established model for prokaryotic transcriptional regulation and provides a novel function for H-NS in preventing the transcription of spurious RNA sequences.

## INSIGHTS INTO BACTERIAL CELL SHAPE, DIVISION, AND DEVELOPMENT

The theme of this session included a range of contributions covering various aspects of bacterial cell biology and cellular development. Lotte Sogaard-Andersen (Max Planck Institute for Terrestrial Microbiology) described her lab's recent studies of the mechanism of cell division in Myxococcus xanthus, an organism well known for activating a multicellular developmental program in response to starvation. She explained that, unlike other model organisms, such as E. coli or Bacillus subtilis, M. xanthus uses a positive regulation mechanism to correctly position its cell division site. Specifically, Lotte described how PomZ, a ParA family ATPase, positively regulates the formation and positioning of the Z-ring, itself made up of the key cell division protein FtsZ (17). Recent data from the group also indicated that PomZ works in concert with two other proteins, PomXY. These three proteins form a cluster associated with the chromosome and are able to dynamically localize to the site of division.

**FIG 3:**
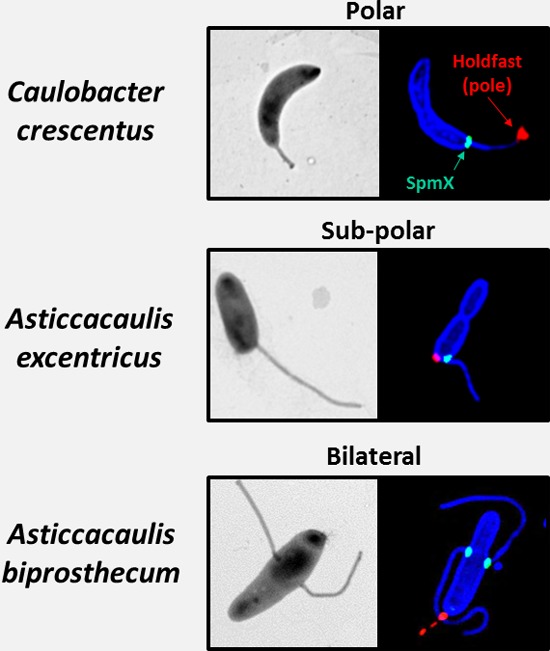
Stalk position and SpmX localization in Caulobacter crescentus (polar), Asticcacaulis excentricus (subpolar), and Asticcacaulis biprosthecum (bilateral). (Left) Electron micrographs of each organism; (right) structured illumination microscopy images. In each case, SpmX is shown in green (SpmX-enhanced GFP), the outer membrane is shown in blue (succinimidyl ester staining), and the holdfast, indicating the pole of the cell, is shown in red (bound to fluorescent lectin). (These images were adapted with permission from Nature [20].)

Two talks continued the theme of development by addressing the fundamental question of how bacterial cells define their shape. Rut Carballido-López (INRA, Jouy-en-Josas, France) highlighted the use of the model organism Bacillus subtilis to gain detailed insight into how the actin-like protein MreB organizes biosynthesis of the peptidoglycan cell wall in order to achieve a stable rod shape. Rut explained how cell wall elongation complexes containing extracytoplasmic peptidoglycan biosynthetic machinery are associated with mobile circumferential patches of polymerized MreB (18). She went on to discuss the lab's recent work using HiLo microscopy, which revealed an association between MreB and the cytoplasmic protein YukR, which is required for peptidoglycan precursor synthesis, suggesting that MreB may coordinate both intra- and extracellular cell wall synthesis machineries (19). Yves Brun (Indiana University), who gave the FEMS-sponsored lecture, continued this conversation by highlighting his lab's use of “exotic” (nonmodel) organisms to gain new insights into the evolution and mechanism of basic cellular processes, including bacterial cell morphogenesis. This point was well illustrated by his group's recent work describing the evolution of stalk positioning in alphaproteobacteria (20). Yves demonstrated that in the genus Caulobacter, the stalk, a distinct morphological appendage involved in nutrient acquisition, is positioned at the cell pole but that in the genus Asticcacaulis, the stalk can be subpolar or bilateral (Fig. 3). Furthermore, the work discovered that a developmental regulator of Caulobacter crescentus, SpmX, has acquired an additional function in Asticcacaulis, where it is necessary to direct stalk synthesis at the appropriate subpolar or bilateral location. Furthermore, a specific C-terminal region of SpmX is required for this positioning function, apparently evolving first to recognize subpolar and then bilateral cell targets.

Another important aspect of bacterial cell biology is the ability to assemble and move proteins across the cell wall and membrane(s). Many presentations at this meeting touched on this area, including the mechanisms, regulation, and roles in virulence of varied protein secretion motility systems. Luke Allsopp (Imperial College London) described the identification of three antibacterial effector proteins secreted by a type VI secretion system in Pseudomonas aeruginosa (21). Each of these effectors is specifically delivered by its own VgrG protein, one of the components of the type VI secretion system involved in penetrating target cells. Lynne Cairns (University of Dundee) described how inhibition of flagellar rotation acts as a mechanical trigger to induce biofilm formation through activation of the DegS-DegU two-component system in B. subtilis (22). This may be a widespread means for motile bacteria to sense that they have made contact with a surface in order to adopt an appropriate developmental program.

## SYMBIOSIS, PATHOGENESIS, AND MECHANISMS OF HOST INTERACTION

The host-microbe interactions—pathogenesis and commensalism session provided an overview of some of the very diverse approaches used to investigate the interplay between bacteria and their hosts. Joan Geoghegan (Trinity College Dublin) presented her work on a group of cell wall-associated surface proteins known as microbial surface components recognizing adhesive matrix molecules (MSCRAMMs), which are important to allow adhesion to and invasion of the host by Staphylococcus aureus as well as evasion of innate immune responses (23). She described her discovery of a subdomain of the N-terminal region of two MSCRAMMs (the clumping factor A and the fibronectin binding protein B), N1, that are required for their export and surface localization.

Changhan Lee from the Römling group (Karolinska Institutet) explained how studies of clone C strains of the opportunistic pathogen P. aeruginosa are particularly prevalent in clinical niches. He detailed how the group originally identified clone C strains and highlighted how these strains contain specific genomic islands containing heat shock- and oxidative-stress-related genes, including the small heat shock protein (sHsp). Taking this further, Changhan described that sHsp confers protection from heat shock and oxidative stress, which may explain the remarkable adaptability of clone C strains to certain clinical settings. Changhan also discussed some of his structure and function data for sHsp, which demonstrated that the protein may be an important holding chaperone. Thibaut Rosay (Université de Rouen) presented his Ph.D. work on the bacterial adaptation to host messengers. Thibaut's team reasoned that during infection of cystic fibrosis (CF) patients, P. aeruginosa may be exposed to the lung hormone CNP (C-type natriuretic peptide). They found that P. aeruginosa responded to *in vitro* exposure to CNP, which appears to lead to a global increase in the intracellular concentration of cAMP in P. aeruginosa and a decrease in the organism's ability to form biofilms (24). They identified AmiC as a potential CNP receptor, and accordingly, an *amiC* mutant strain formed less biofilm. Thibaut is currently examining the interaction between CNP and AmiC.

To evade the host immune responses, some intracellular pathogens have developed means to directly survive within host phagocytic cells. Miguel Valvano (Queen's University Belfast) described how the opportunistic intracellular pathogen Burkholderia cenocepacia takes advantage of the autophagy defect of macrophages in CF patients. B. cenocepacia survives but barely replicates within proficient phagocytic cells. After internalization, the bacteria translocate type VI secretion system effectors, which interfere with the actin cytoskeleton and contribute to host cell pyroptosis. Type VI secretion also leads to an alteration of the phagosome membrane that encloses the bacteria during activation of autophagy. Autophagy thereafter limits bacterial survival, but in defective macrophages of CF patients, autophagy is compromised. In these cases, B. cenocepacia further compromises autophagy and therefore can survive better. Miguel demonstrated that autophagy-stimulating drugs helped to clear B. cenocepacia from the macrophages (25). Sophie Helaine (Imperial College London) presented her work on Salmonella enterica serovar Typhimurium, an intracellular model pathogen. By developing a method (fluorescence dilution) to monitor bacterial intracellular replication at the single-cell level, the Imperial College team showed that after phagocytosis by host macrophages, part of the Salmonella population survived in a nonreplicating state. These nonreplicating bacteria can then tolerate high doses of antibiotic both in *in vitro* cellular models and in infected mice and are likely to be a cause of recurrent infections. Sophie showed the first direct evidence for the presence of nonreplicating persister bacteria during host infection (26).

**FIG 4:**
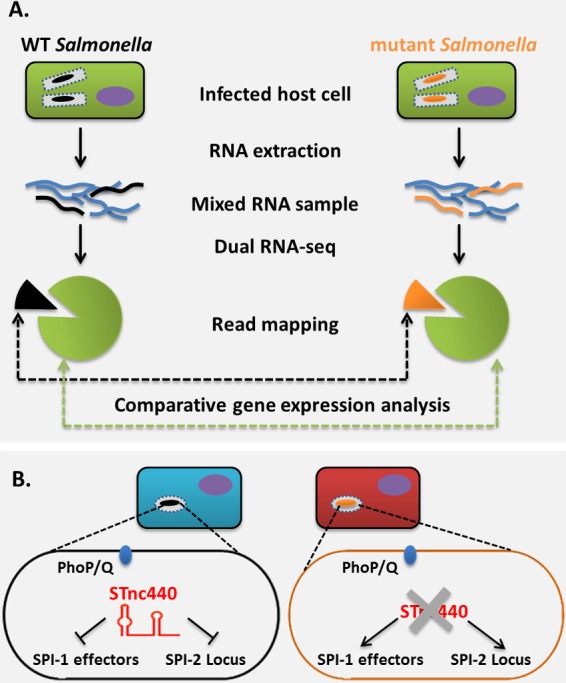
Dual RNA-Seq of Salmonella Typhimurium in epithelial cells. (A) After infection of epithelial cells with wild-type (WT; black) or STnc440 mutant (orange) strains of Salmonella Typhimurium, changes in gene expression were simultaneously compared for both the host and pathogen by dual RNA-Seq. (B) STnc440 is a PhoP-dependent sRNA that is highly activated in intracellular Salmonella and is required for the timely repression of Salmonella SPI1 and SPI2 virulence factors to avoid a hyper-inflammatory response (red) from the host.

Both Marcus Claesson (University College Cork) and Alexander Westermann (Institute for Molecular Infection Biology, Würzburg, Germany) presented pioneering dual-transcriptome sequencing (RNA-Seq) approaches to study very different aspects of bacterial pathogenesis (Fig. 4). With RNA-Seq, full transcriptomes can be analyzed without the bias of probe-dependent approaches, such as microarrays (27). Marcus focused on how the intestinal microbiota influences inflammatory bowel diseases (IBD), such as ulcerative colitis (UC) and Crohn's disease (CD). By using high-throughput RNA-Seq, Marcus's team assessed the microbial gene expression in inflamed and noninflamed mucosa biopsy specimens collected from 6 UC and 6 CD patients in a pilot project. Thus, rather than presenting just a view of the microbiota composition, they questioned the types of genes and potential functions expressed by the microbiota during healthy and diseased tissue colonization. Patients of the two diseases clustered differently, more so for the human than for the bacterial transcriptomes. A future project will comprise a larger sample size and will also include information on diet, host genotype, and medication and should enable the identification of subcohorts of patients with these diseases, as well as correlated microorganisms.

Alexander demonstrated that with dual RNA-Seq, the Vogel group has achieved a further step forward in transcriptomics and the analysis of host-pathogen interplay (Fig. 4). After infection of epithelial cells with Salmonella Typhimurium, changes in gene expression were simultaneously monitored for both host and pathogen in an attempt to relate a given action with its respective reaction. Among numerous infection-associated gene expression changes, Alexander focused on a bacterial small RNA, STnc440, that is highly induced once Salmonella becomes intracellular. A second round of dual RNA-Seq provided a time course comparison of transcriptome changes of the wild-type strain and STnc440 mutant strains as infection was progressing. This very detailed work revealed that STnc440 was required for the timely repression of Salmonella virulence factors to avoid a hyper-inflammatory response from the host.

## BACTERIALLY COORDINATED BEHAVIOR, INTERACTIONS, AND COMMUNICATION

The final session covered the topic of microbe-microbe interactions, which included talks on microbial antagonism, interactions within communities, and biofilm formation. Mervyn Bibb (John Innes Centre) kicked off the session by discussing his group's work on the regulation of antibiotic production in Actinomycetes. He described their recent findings on the complex system involved in the regulation of the biosynthesis of the lantibiotic microbisporicin in Microbispora species and the important role of antibiotics in potentially shaping the microbial communities in which they are produced. Mervyn explained how the group was originally able to identify a cluster of 20 genes required for the production of microbisporicin and to show that three genes, *mibX*, *mibW* (which encode a sigma factor and membrane-associated anti-sigma factor, respectively), and *mibR*, were involved in the regulation process (28, 29). The MibX and MibW proteins were shown to interact using a bacterial two-hybrid assay and to constitute a feed-forward system for the regulation of microbisporicin biosynthesis and immunity that is triggered by the initial expression of *mibR* (28). His group was also able to show that microbisporicin and other actinomycete lantibiotics can act as signaling molecules to trigger and presumably coordinate their own biosynthesis throughout the producing population.

**FIG 5:**
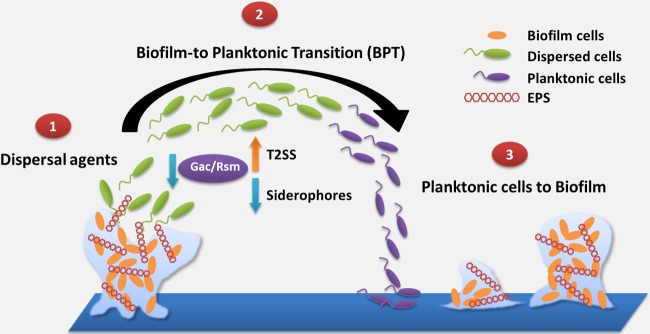
Model of the biofilm-planktonic life cycle, with dispersed cells as a unique intermediate between the planktonic and biofilm lifestyles. (1) The addition of dispersal agents causes motile cells to disperse from the biofilm (shown in green). (2) Dispersed cells have lower expression of the Gac-dependent small RNAs RsmY and RsmZ, higher expression of T2SS, and lower production of siderophores, such as pyoverdine, than planktonic cells (shown in purple). (3) Dispersed cells revert to the planktonic mode, and planktonic cells eventually colonize new surfaces and form biofilms. For further details, see reference 30.

Other excellent talks were given by amazing young speakers, as described above. Lynne Cairns (University of Dundee) and Natalia Tschowri (HU-University of Berlin) highlighted important signaling aspects of microbe-microbe interactions, while Liang Yang (Nanyang Technological University) presented his work on bacterial interactions during mixed-species biofilms. Liang discussed his postdoctoral work using a flow cell biofilm model to demonstrate that cell clusters of S. aureus can trap P. aeruginosa during the early stages of biofilm formation and that the interaction is mediated by type IV pili and extracellular DNA (30). Using a *cdrA*-green fluorescent protein (GFP) fusion to report on c-di-GMP cellular levels, he showed that c-di-GMP levels increase in P. aeruginosa during mixed-species biofilm formation. Liang also presented recent data by his own laboratory showing that it is possible to interfere with c-di-GMP signaling to disperse such biofilms but that dispersing cells tend to be more virulent (Fig. 5). Thus, cyclic di-GMP addition may potentially be used to control biofilm formation, but antibiotics would have to be used in conjunction to prevent subsequent infections.

As mentioned above, earlier in the meeting, Liz Sockett (University of Nottingham) presented her group's work on the role of c-di-GMP in the control of prey invasion by Bdellovibrio bacteriovorus. Liz described how c-di-GMP is known to control the switch between predatory and nonpredatory life cycles by controlling several enzymes required for the prey invasion and degradation process. Interestingly, Bdellovibrio possesses 7 proteins predicted to be involved in c-di-GMP turnover and more than 15 proteins involved in binding the dinucleotide (31). Liz highlighted one of the group's published stories showing that CdgA, an inactive degenerate GGDEF domain protein, was able to localize at the interaction pole and to be required for prey invasion (31). Liz's group recently extended this study to demonstrate that CdgA forms complexes with other proteins, similar to those seen in Myxococcus to control cell polarity and type IV pilus-mediated gliding motility (32). Unlike in Bdellovibrio, the CdgA protein interacts with MglA and/or RomR to form a complex required for prey invasion (32). The role of c-di-GMP in this complex formation still remains to be elucidated.

## CONCLUDING REMARKS

In terms of science, this symposium lived up to the expectations from the previous meetings (1, 2). Again it provided an excellent forum for those with an interest in microbiology to interact and gain exposure to a wide range of topics. The animated and exciting discussions between senior academics and scientists who are just beginning their careers during the oral and poster sessions clearly indicated the quality of work on display.

Since this was the third YMS and little is known about the direct impact of attendance, we carried out a retrospective analysis with past participants and attendees. The quantitative research consisted of questionnaires completed by students and postdocs (181 in total) that participated in the previous meeting(s). The qualitative research took the form of semistructured interviews with previously invited postdocs or students (33 in total) who gave talks at a previous meeting(s). In the approaches employed (interviews, questionnaires), the emphasis was on the students' and the postdocs' perceptions of the nature of the YMS meeting, its purpose, benefits, and outcomes. The findings from the analysis make it clear that from the point of view both of students and of postdocs participating, the YMS has a wide range of significant positive benefits, including opportunities to enhance personal attributes and qualities, opportunities to associate with others in a positive context, opportunities for new and more diverse experiences and for improving practical skills, access to information, and exposure to advice and advocacy in relation to education and career development. The analysis revealed that 42% (*n* = 24) of invited postdoc speakers have made the transition to PI positions since the year that they spoke at the meeting and that 54% of the Ph.D. students that attended the meeting have gone on to gain a postdoc position or fellowship advertised at the meeting, outcomes directly related to a young scientist's career development.

After the final session, a number of awards were distributed by Doreen Cantrell (University of Dundee). These included a poster talk prize, sponsored by Biochemical Journal, which was given to Valerie O'Brien (Washington University School of Medicine) for her presentation entitled “Prior Infection Alters the Bacterial Requirement for Virulence in a Mouse Model of Recurrent Chronic Bladder Infection.” The poster prize, which was sponsored by Molecular Microbiology, went to Stéphanie Borland (University of Lyon) for her poster entitled “Role of Phosphorelays in Azospirillum: from Genomics to Functional Analysis.” Stéphanie's work was selected from over 80 posters. Besides the various scientific highlights of this meeting, there were some outstanding social events, including a conference dinner overlooking the river Tay and a traditional Scottish ceilidh. The feedback from attendees was very positive; participants appreciated the quality of the scientific program and the intimate atmosphere of a small conference. Given the great amount of enthusiasm for the venue and the event, another meeting will be held at the University of Dundee in 2016.
